# Good and Magical Times

**DOI:** 10.31486/toj.21.5019

**Published:** 2021

**Authors:** Ronald G. Amedee

**Affiliations:** Head of Clinical School and Professor, The University of Queensland Faculty of Medicine, Ochsner Clinical School, New Orleans, LA; Editor-in-Chief, *Ochsner Journal*

*Everything good, everything magical, happens between the months of June and August.*

*–Jenny Han*

Seven original research articles, one quality improvement paper, one contemporary review/update, and ten case reports/clinical observations serve as the principal elements of the summer edition of the *Ochsner Journal*.

Regarding original research, “Personality, Resilience, and Calling in Students Undertaking a Medical Degree Across Two Continents: Disparate Pathways to the Healing Profession” by Galarneau, Seoane, and Eley describes essential personality traits that have led to success in The University of Queensland-Ochsner Clinical School student cohort. Another interesting read by Dahmash, Alajmi, Aldayel, et al is “Burnout and Associated Risk Factors in Pediatric Residents,” which is a particularly enlightening manuscript to review during our current point in the COVID-19 pandemic recovery.

The quality improvement paper for this edition is authored by Gupta VK, Gupta KK, Sanghera, et al and details “A Basic Intervention to Improve Compliance with Thromboprophylaxis in Patients With Neck of Femur Fracture.” Groff, Kavanaugh, Ramgobin, et al contribute the issue's review article, “Gastrointestinal Manifestations of COVID-19: A Review of What We Know.”

The case reports/clinical observations manuscripts include “Iatrogenic Middle Cerebral Artery Ruptured Pseudoaneurysm Successfully Treated With a Pipeline Embolization Device,” submitted by Scullen, Mathkour, Carr, et al, and “Heparin-Induced Thrombocytopenia After Mitral Valve Replacement” by Tuglan, Chang, and Bates.

This edition also contains our quarterly Sports Medicine column, “It's Not De Quervain Tenosynovitis – A Diagnosis to Consider in Persistent Wrist Pain” penned by Schmidt, Kobayashi, and Gottschalk.

One good and magical thing occurred as I wrote this introduction: the recent announcement of relaxed CDC guidance that fully vaccinated folks no longer need to wear masks in many places except in patient-facing situations such as in hospitals/medical clinics and onboard a commercial aircraft. Our state has administered nearly 3.1 million doses of vaccine, and presently 1.3 million Louisiana citizens are fully vaccinated. However, work remains in our vaccination efforts as Louisiana is home to more than 4.9 million citizens.

Since we published the Spring 2021 edition of the *Journal*, Ochsner Health lost one of our legacy physicians with the passing of Dr Francis E. (Duke) LeJeune, Jr. (see inside front cover for photograph). Duke was a kind-hearted southern gentleman who was small in stature but hugely impactful on the specialty of Otolaryngology–Head and Neck Surgery and the Ochsner Clinic that his father helped to found. He succeeded his father in 1963 as the department chair at Ochsner and served with distinction until he retired in 1996. During that time, he helped to train and educate the next generation of specialists, several of whom have either worked or are still employed at Ochsner. I guess you could say he was a “gentle giant,” a “servant leader,” and a “valued supporter” of Ochsner Philanthropy along with his wife Kay who survives him. He will be sadly missed by all of us who knew and loved him.

